# Detection, quantification, and characterization of airborne *Aspergillus flavus* within the corn canopy

**DOI:** 10.1007/s12550-025-00581-6

**Published:** 2025-01-14

**Authors:** Mark A. Weaver, Lilly C. Park, Michael J. Brewer, Michael J. Grodowitz, Hamed K. Abbas

**Affiliations:** 1 ARS, National Biological Control Laboratory, 59 Lee Road, Stoneville, MS 38776 USA; 2 Entomology Program, Texas A&M AgriLife Research & Extension Center, 10345 State Hwy 44, Corpus Christi, TX 78406 USA

**Keywords:** Mycotoxin, Maize, Aerobiology, Aflatoxin, Conidia, Small sclerotia

## Abstract

Aflatoxin contamination of corn can occur when developing kernels are infected by the plant pathogen *Aspergillus flavus*. One route of infection is from airborne conidia. We executed a series of experiments within the corn canopy during two growing seasons and in two states to document the abundance and dynamics of the airborne *A. flavus* population. We did not observe any significant diurnal changes in the conidial density (*p* = 0.171) or any effect of sampler height (*p* = 0.882) within the canopy. Significant changes (*p* < 0.001) were noted during the season, with a trend towards increased airborne populations with later stages of corn development and more than a 20-fold increase from July to August. The median aflatoxigenicity of airborne isolates from a corn canopy in Texas was about 50 times higher than the corresponding population in Mississippi. It was also noteworthy that highly aflatoxigenic, weakly sporulating S-morphotypes accounted for 14–30% of the airborne isolates in Mississippi at a site with historically rare abundance of S-morphotypes. The genetic diversity was high among the 140 analyzed airborne isolates, with 76 unique haplotypes identified and 55 haplotypes occurring only in 1 isolate. Even in the context of this highly diverse population, a haplotype matching that of a commercial biocontrol strain was found in 13 of the 70 isolates from Mississippi and 1 of the 70 isolates from Texas. The airborne *A. flavus* population is genetically diverse (Shannon’s index = 1.4 to 1.6), similar to grain samples in other surveys, and much less aflatoxigenic in Mississippi than in Texas.

## Introduction

The United States planted 38 million hectares (94 million acres) of corn in 2023 (USDA-NASS 2024). One of the many production challenges that growers face is late-season infection of grain by the opportunistic pathogen *Aspergillus flavus*. These infections generally do not cause a measurable yield loss, but the infections are economically important because growth of the fungus is often accompanied by the accumulation of aflatoxin (AF). AFs are group of related secondary metabolites, including aflatoxin B1, the most potent carcinogen found in nature (Wogan 1992). The dangers of AF are widely recognized for humans (Heinrich 2003) and animals (Popescu et al. 2022), so there are regulations for AF levels in food and feed. In developed nations, extensive testing is performed to exclude AF from the food chain (Krska et al. 2012). In addition to the indirect production costs of AF contamination in the form of testing and mitigation efforts, the direct economic loss in the US may be up to US $1 billion annually (Mitchell et al. 2016).

The signs and symptoms of *Aspergillus* ear rot of corn are easily recognized and were well described over 100 years ago (Taubenhaus 1920). These visible infections are often associated with insect injury to kernels, especially from larval feeding of corn earworm, *Helicoverpa zea* (Boddie), and fall armyworm, *Spodoptera frugiperda* (J. E. Smith) (Lepidoptera: Noctuidae) (Dowd et al. 2005; Pruter et al. 2020). But infections also occur in the absence of apparent insect activity, and *A. flavus* is known to directly infect kernels (Diener et al. 1987). Infections in the absence of insect activity are presumably through airborne propagules of *A. flavus*. Microscopic examination of airborne spores is challenging because the spores are not easily differentiated from other airborne particles (Bock and Cotty, 2004). DNA-based detection methods are possible, but direct enumeration on selective media is also simple, convenient, and affordable if the collection method facilitates collection of viable propagules. Some strains of *A. flavus*, the S-morphotypes, are strongly aflatoxigenic and produce abundant, small sclerotia (< 400 μm), and very few conidia. Other strains, the L-morphotypes, are variable in their aflatoxigenicity and produce very few, large sclerotia (> 400 μm) but up to 60 × more conidia (Cotty 1989; Sweany et al. 2024). The conidia of *A. flavus* are small (less than 10 µm), dry, and readily dispersible in the air. It has previously been reported that genotypes of S-morphotypes of *A. flavus* which are somewhat common in soil are very rarely found on corn (Sweany et al. 2011, 2024). The rare infection of corn by S-morphotypes from the soil could be a result of low virulence on corn, or it could be because the S-morphotypes are producing such a limited amount of air-dispersible inoculum that can reach the developing corn ears.

We are interested in the transmission of *A. flavus* in the corn agroecosystem and parsing the roles and relative importance of insect transmission and deposition of airborne propagules in the infection process. While there are other accounts of detection of airborne *A. flavus* propagules in an agricultural context (Bock and Cotty 2006), we are specifically interested in the detection and characterization of airborne *A. flavus* within the canopy of corn at a time period when corn is susceptible to infection by *A. flavus*. The objective of the present research is a quantitative (numerical) and qualitative (chemotype, morphotype, haplotype) description of the airborne *A. flavus* population in corn fields at a time when corn is susceptible to *A. flavus* infection. We describe here a replicated measurement of the airborne population of *A. flavus* at two time points and at two elevations in the corn canopy, the observation of a highly aflatoxigenic morphotype in air samples, and differentiate 76 unique haplotypes in 140 isolates.

## Materials and methods

### Air sampling methods

**Fig. 1:**
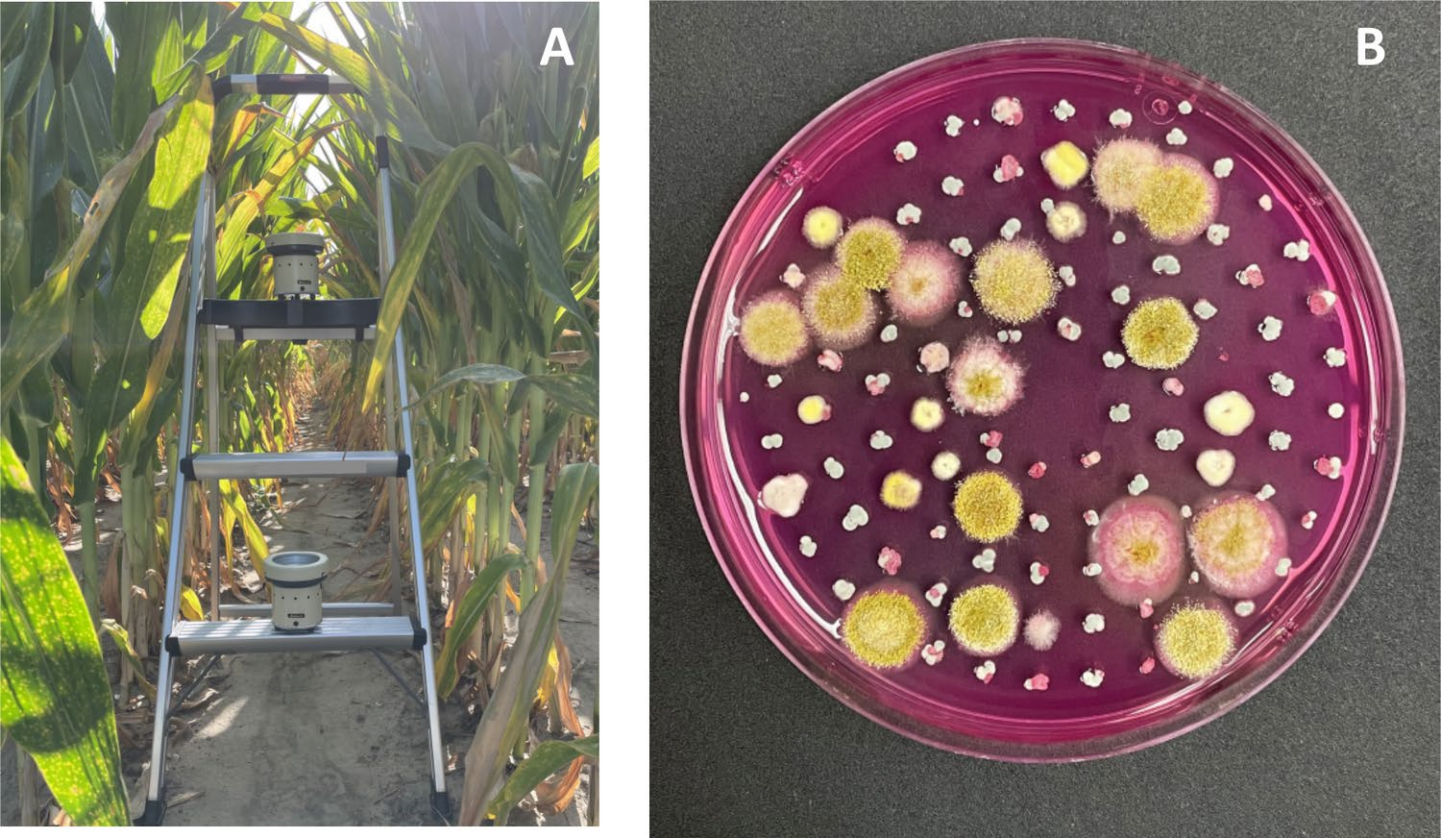
**A** Air sampling system. Burkard portable air sampling unit placement within the corn canopy with sampling units at 0.3- and 0.9-m elevation. **B** Example of deposition of airborne propagules on mDRB selective growth medium

In 2022, air sampling was conducted within corn research fields at the Stoneville, Mississippi site, and near Corpus Christi, TA, USA; planted in 102-cm rows with hybrid NK1694-3111 and DK65-95, respectively, with conventional tillage, preplant fertilizer at both sites; and irrigated with drip lines (TX) and furrow (MS) as needed. Air samples were collected when the corn was at growth stages R3–R4 (“milk” to “dough”) on June 15 in Corpus Christi with a Burkard cyclone air sampler and at R4 on July 19–20 at Stoneville with a Burkard portable air sampler placed approximately 20 m from the field edge. The experiment was repeated at both sites in 2023, with hybrids 62–08 and 65–93 and Stoneville and Corpus Christi, respectively. R3 stage air sampling was conducted at Corpus Christi on June 15, 2023, with the Burkard portable air sampler. The Stoneville site was sampled more intensively in 2023, from July 6 to 11, 2023, in R4 corn and again from Aug 10 to 16 in R6 (mature) corn. For the 2023 Stoneville sampling, the monitoring was conducted continuously, day and night, unlike the approximate 10 h per day at other dates. At the Stoneville location in 2023, Burkard portable air samplers were placed in the field at two elevations, 0.3 m and 0.9 m (Fig. 1A) to evaluate potential differences in airborne propagules near the ground and at approximately the height of corn ears. Meteorological observations were collected from the National Oceanic and Atmospheric Administration site Corpus Christi station, 6 km from study site (https://www.ncei.noaa.gov/cdo-web/datasets/GHCND/stations/GHCND:USW00012924/detail), and Mississippi State station AWS (http://deltaweather.extension.msstate.edu/stoneville-aws), 2 km from study site.

## Microbiological observations

The Burkard portable air samplers used at both dates in MS and in 2023 in TX stream the airflow directly onto selective modified Dichloran Rose-Bengal media (mDRB) (Horn and Dorner 1998) (Fig. 1B). The Burkard cyclone sampler used in 2022 in TX collects the sample in sterile tubes. Those samples were diluted with 0.1% Triton water and plated on mDRB. After 2–3 days of incubation at 37 °C, the number of *A. flavus* colonies was counted, and the colony-forming units (CFU) at each sample date and elevation were analyzed by ANOVA. Individual colonies were streaked on clean mDRB to ensure pure isolates.

## Analysis of airborne isolates

Individual isolates were transferred to potato dextrose agar. After 7 days’ incubation at 28 °C, the plates were visually inspected to classify isolates as L- and S-morphotype based on the abundance of sporulation and the presence, size, and abundance of sclerotia (Cotty 1989; Sweany et al. 2024). Sclerotia were directly visualized and measured on a Keyence VHX Digital Microscope with VH-Z20R/W/T objective at 150 × magnification. Sporulating agar plugs were transferred to screw top vials with sterile water for long-term storage. To determine aflatoxigenicity, agar plugs were transferred to 50-mL sterile conical tubes with gas-permeable lids and 5 g of cracked corn, adjusted to 25% moisture. After 1 week of growth at 28 °C, 10-mL 0.1% Triton X-100 was added and mixed to dislodge spores. One milliliter of this spore suspension was removed for DNA extraction, and 20 mL of methanol was added to the remainder to extract aflatoxin. This methanolic extract was passed through a 0.2-µm filter and quantified via HPLC with fluorescence detection, aided by post-column UV photochemical derivatization (Weaver et al. 2022). The DNA was purified by the method of Callicott et al. (2018). Simple sequence repeats (SSRs) were amplified with 16 primer pairs (Grubisha and Cotty 2009, Weaver et al. 2022, Weaver et al. 2023). Amplicons were separated on an ABI 373 DNA Analyzer with the LIZ 500 size standard and scored with Geneious Prime v. 2024.0. Haplotypes were entered into GenoDive v 3.06 (Meirmans 2020; Meirmans and Van Tienderen 2004) to identify clones (threshold set at 1) and determine genetic distances via Nei’s test and processed with GenAlEx v6.5 (Peakall and Smouse 2012) for principal coordinate analysis (PCoA) and calculations of genetic diversity.

## Results

### Monitoring of *A. flavus* in the corn canopy in Corpus Christi, TX, USA (2022–2023)

**Fig. 2:**
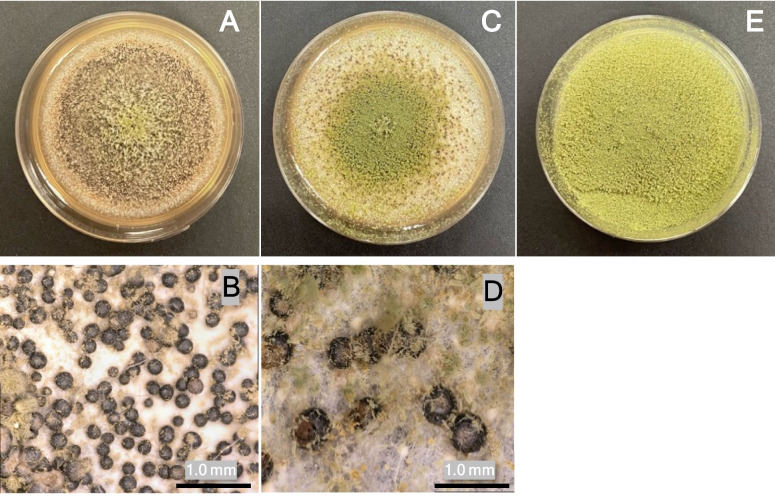
Representative *A. flavus* morphotypes grown for 1 week in the dark at 28 °C on potato dextrose agar. **A** and **B** are examples of an S-morphotype isolate. Note the abundance of small dark sclerotia (198 µm + / − 9 µm and limited sporulation. **C** and **D** are examples of L-morphotypes with prolific sporulation and a limited number of large sclerotia (435 µm + / − 15 µm*)*. Many L-morphotype strains do not produce any sclerotia, as in **E**. One-millimeter scale bar given for reference

Characterization of the first 50 isolates from Texas in 2022 and 2023 from R4 stage corn is presented in Table 1. About 10% of the recovered airborne isolates were the highly aflatoxigenic S-morphotypes (Fig. 2) with an average sclerotia size of 198 µm + / − 9 µm. The aflatoxigenicity of these isolates, when grown in vitro on autoclaved corn, is presented in Table 1. For the 100 isolates, over the 2 years of observations, there were 13 nonaflatoxigenic isolates and 43 highly aflatoxigenic isolates, producing greater than 300 µg/kg AF on autoclaved corn.

**Table 1:** Morphotype and chemotype of *A. flavus* isolates collected in 2022 and 2023 from Texas and Mississippi. The first 50 isolates from each site-year were characterized based on the size and number of sclerotia produced in vitro and the amount of aflatoxin produced on cracked corn

	Number of isolates					
		Aflatoxigenicity				
	S-morphotype	Zero	Low < 20 ppb	Medium 20–299 ppb	High > 300 ppb	Median toxigenicity
Texas 2022	6	6	13	8	23	135 ppb
Texas 2023	5	7	11	12	20	92 ppb
Mississippi 2022	15	13	14	9	14	2 ppb
Mississippi 2023	7	17	12	5	16	2 ppb

### Monitoring of *A. flavus* in the corn canopy in Stoneville, MS, USA (2022–2023)

**Fig. 3:**
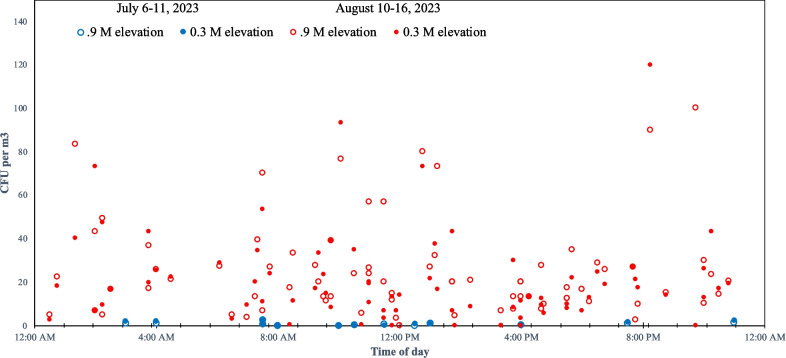
Effect of elevation and time of day on airborne spore concentration as measured in July (R4-stage corn) and August (R6-stage corn) at Stoneville MS. Burkard portable air samplers were positioned at 0.3-m (closed circles) and 0.9-m (open circles) elevation in the corn canopy to collect airborne *A. flavus* spores. No significant effect was observed for time of day or elevation, but the observed spore concentrations were more than 20 × greater in August (in red) than July (blue)

Characterization of the first 50 isolates from Mississippi in 2022 and 2023 from R4 stage corn is also presented in Table 1. S-morphotypes comprised about 20% of the airborne isolates in this study. While all of these S-morphotypes are highly aflatoxigenic, most airborne isolates were non-aflatoxigenic or produced less than 20 µg/kg ppb AF on autoclaved corn. To further explore the spatial and temporal dynamics of the airborne *A. flavus* population, replicate air samplers were placed in the corn canopy at 0.3- and 0.9-m elevation and operated periodically in R4 corn (July 6–11, 2023) and continuously for 1 week in mature corn (R6 growth stage). The spore density within the corn canopy increased from 1.1 CFU per m^3^ in the R4 corn to 23 CFU per m^3^ in R6 stage corn (Table 2 and Fig. 3). This 20-fold increase was statistically significant (*p* < 0.001) (Table 3). Local weather conditions during the air sampling period and the 10 days preceding these periods are also reported in Table 2 and are broadly similar, with the notable difference in wind speed with Corpus Christi, TX, USA, being much windier than Stoneville, MS, USA.

**Table 2:** Airborne *A. flavus* spore density and meteorological observations in 2023. All *A. flavus* isolates collected with Burkard portable air samplers, as seen in Fig. 1

		Avg. temp. (°C)		Humidity (%)		Avg. daily precipitation	Avg. wind	Observed *A. flavus*
		Max	Min	Max	Min	mm	km/H	CFU/m^3^
Texas R3–4 stage (June 15, 2022)	Monitoring period	33	26	90	63	0	26	Not quantified
	10 days preceding	34	24	94	61	0	26	
Texas R4 stage (June 15, 2023)	Monitoring period	34	23	100	60	2	15	2.8
	10 days preceding	35	26	100	55	0	21	
Mississippi R4 stage (July 6–11, 2022)	Monitoring period	34	25	89	52	0	8	Not quantified
	10 days preceding	34	23	99	62	0	4	
Mississippi R4 stage (July 6–11, 2023)	Monitoring period	35	23	99	64	1	8	0.75
	10 days preceding	32	22	98	60	4	6	
Mississippi R6 stage (Aug 10–16, 2023)	Monitoring period	35	23	96	51	1	10	23
	10 days preceding	36	24	95	52	7	10

**Table 3:** Analysis of variation for 2023 Mississippi air sampling results. Test of elevation and time of day as main effects (two and four levels, respectively) and interaction

ANOVA						
Source of variation	SS	df	MS	*F*	*p*-value	
Time of day	0.0009	3	3.08E-04	1.708	0.171	
Elevation	3.97E-06	1	3.97E-06	0.022	0.882	
Interaction	0.0001	3	3.39E-05	0.188	0.904	
Within	0.0158	88	1.80E-04			
Total	0.0169	95				

### DNA-based characterization of airborne *A. flavus* isolates from the corn canopy

The SSRs used here revealed high genetic diversity within these 140 isolates (35 individual isolates × 2 locations × 2 years). Each locus identified between 4 and 23 alleles and combined to differentiate 76 unique haplotypes (Table 4). Even with this high diversity, 1 haplotype was shared by 13 MS isolates and 1 TX isolate. This haplotype is identical to that of *A. flavus* strain 21,882, which is commercially available as the biocontrol product Afla-Guard. The haplotype of the other commercially available biocontrol isolate used in the US, AF36 Prevail, was found in 2 of the 70 TX isolates but not found in MS.

**Table 4:** SSR amplicons of airborne *A. flavus* isolates and two commercially available biocontrol isolates. The most common isolate from MS is identical to strain 21,882. Eleven alleles are shared between the most common haplotypes from MS and TX and strain 21,882 (shaded cells)

							**SSR loci**									
	AF28	AF13	AF22	AF31	AF53	AF34	AF42	AF8	AF16	AF54	AF17	AF11	AF66	AF64	AF63	AF55
No. of unique alleles	12	15	9	17	10	13	17	19	10	6	12	19	10	23	4	14
Most common MS haplotype	119	141	144	312	131	296	146	168	169	161	353	138	269	161	127	180
Most common TX haplotype	119	128	144	312	131	296	146	180	169	161	362	138	269	163	127	172
21882 haplotype	119	141	144	312	131	296	146	168	169	161	353	138	269	161	127	180
AF36 haplotype	119	161	188	309	134	310	162	177	191	169	353	162	269	211	135	174

**Fig. 4:**
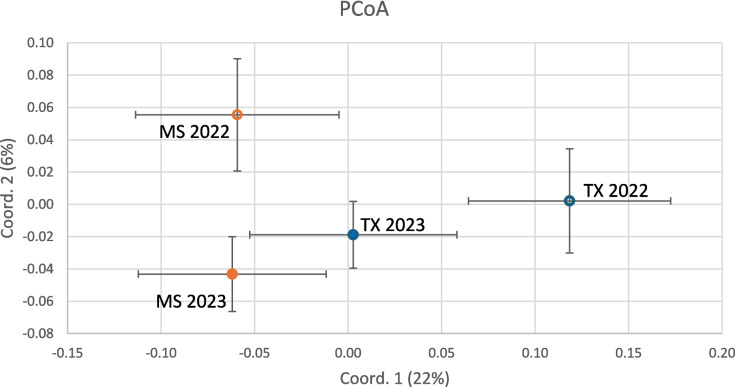
Principal coordinate analysis of Mississippi and Texas airborne *A. flavus* isolate haplotypes. Thirty-five isolates from each of the 4 site-years were genotyped by SSR and separated by PCoA

**Fig. 5:**
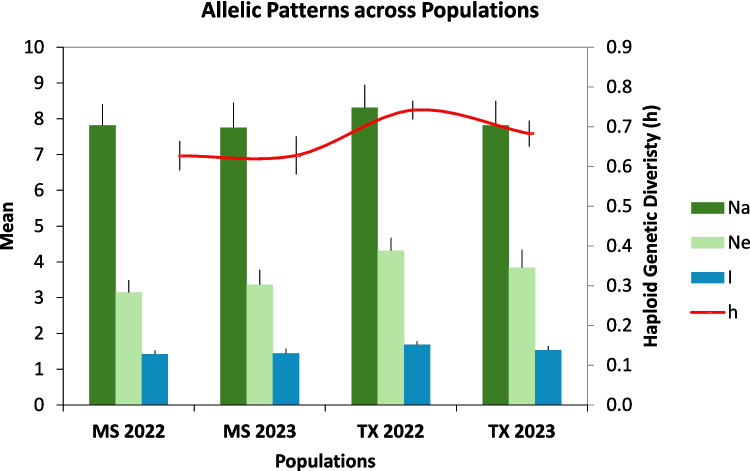
Diversity indices for airborne *A. flavus* populations. Na is the number of alleles. Ne is the number of effective alleles. Haploid genetic diversity is given as *h*. Shannon’s information index is given as *I*. Sample size is 35 for each population

Measurements of the population-level genetic diversity are given in Table 4 and Fig. 4. The genetic distances between the four populations, as calculated by Nei’s statistic and PCoA, are presented in Table 5 and Fig. 5. The two MS populations (2022 and 2023) had a small degree of separation overall (distance 0.003) but were resolved by axis 2 of the PCoA. The 2022 TX population was the most distinct, with a separation of at least 0.037 from all other populations.

**Table 5:** Nei’s test of genetic distance between populations. The Texas 2022 population was the most genetically distinct, with the greatest separation from all other populations

Population	Mississippi 2022	Mississippi 2023	Texas 2022	Texas 2023
Mississippi 2022	0	0.003	0.054	0.031
Mississippi 2023	0.003	0	0.048	0.028
Texas 2022	0.054	0.048	0	0.037
Texas 2023	0.031	0.028	0.037	0

## Discussion

Previous research explored the airborne *A. flavus* population dynamics in the context of aflatoxin biocontrol. That work and the present research had important differences, but several of the same results (Bock et al. 2004) found abundant S-morphotypes in their air samples, consistent with the knowledge that S-morphotypes were common in that region (Southwestern Arizona, USA). The discovery of S-morphotypes in our Mississippi samples, however, was more surprising because those isolates have not been commonly recovered in Mississippi and the Mississippi River alluvial region (Horn and Dorner 1998, Sweany et al. 2011) and are relatively uncommon at this site (Weaver et al. 2015, 2019). The fact that S-strains sporulate so poorly (Sweany et al. 2024) makes it likely that air sampling will underestimate their abundance.

In 1975, Anderson et al. helped to prove that AF infection of corn is not just a postharvest phenomenon by demonstrating infection of corn, in the absence of insect damage, occurring from 2 weeks after silking until the mature kernels dried to below 20% moisture. We are interested in the *A. flavus* population in the corn canopy during this window of susceptibility so that we can better understand the sources of inoculum (local or regional), the roles of insect in *A. flavus* infection, and the potential for niche specialization within *A. flavus*. Air sampling has been conducted with low-level air samplers (Bock et al. 2004) and with samplers at approximately ear height (Jones et al. 1981). We monitored at both 0.3-m and 0.9-m elevation and detected no significant differences. Plausible models for corn infection by *A. flavus* might suggest dispersal to the ear directly from an *A. flavus* spore plume, emerging from soil level, but we did not observe a decrease in spore concentration at the higher elevation, as presumed by this conceptualization. Instead, our observation of similar airborne spore concentrations at both elevations in our 2023 MS samples would indicate more possibilities for regional dispersal and mixing of the population across the landscape.

The TX population in both years of this experiment had a greater than 50 × median aflatoxigenicity value compared to the MS population, along with less than half as many non-aflatoxigenic isolates. We have previously observed that AF contamination of corn is much more frequent and severe at the TX location than the MS location (Weaver et al. 2017). There are several weather- and soil-based factors that likely contribute to the differences (e.g., Castano-Duque et al. 2024), but the differences in the local *A. flavus* populations could also be contributors. When genetically characterizing the *A. flavus* populations on commercial corn samples from the Southeastern United States, we noted that about half of the *A. flavus* isolates had a 21882-like haplotype (Weaver et al. 2022). We have previously noted the abundance of 21882 or 21882-like *A. flavus* at this location and throughout the MS Delta (Weaver et al. 2019; Weaver and Abbas 2019; Chang et al. 2020). The 21882 commercial product has not been applied at MS site of the current experiment, but it appears that this haplotype is well adapted to the area and is a major part of the local *A. flavus* population. In contrast, that haplotype was only seen in 1 of the 70 TX isolates. It is possible that locally adapted, indigenous, non-aflatoxigenic *A. flavus* populations are providing significant protection against AF contamination at the MS location. While there are numerous physical and climatic differences between the TX and MS sites, the differences in the *A. flavus* population could be contributing to make TX corn more prone to aflatoxin contamination.

Examination of the genetic distances between the four populations (Table 5 and Fig. 5) reveals differences that suggest differentiation between the two sites and between the 2 years. The diversity metrics are like those reported in other *A. flavus* populations using the same SSRs (Weaver et al. 2022, 2023), and the four populations observed here were similarly diverse. While there is no evidence to point to the mechanism for the genetic divergence, the population differences could have implications for aflatoxin management in corn. For example, these population differences could contribute to the well-documented genotype × environment interactions in aflatoxin resistance in corn (Okoth et al. 2017).

In addition to the work in an agricultural context discussed above, there is great interest in the airborne dispersal of *A. flavus* by the medical and occupational safety disciplines. *A. flavus* can be a dangerous human pathogen, and even nonviable spores can be medically significant allergens (Hedayati et al. 2007). While *Aspergillus fumigatus* is the most important *Aspergillus* species for sinus and pulmonary infections, *A. flavus* is the second most medically important *Aspergillus* species and the most common cause of cutaneous aspergillosis and non-respiratory infections (Hedayati et al. 2007). Nosocomial outbreaks can sometimes be linked to seasonal or temporary spikes in detection of airborne spores (Falvey and Streifel 2007, Hajjeh and Warnock 2001). There are some parallels between human aspergillosis and *A. flavus* infection of corn: The infection and the subsequent outcomes are strongly conditioned by the antecedent health of the host. The likelihood of an infection may be predicated by the quantity of airborne spores during windows of vulnerability, and minimizing exposure to airborne spores may be a factor in avoiding infections.

Corn growers weigh competing factors in their management decisions. For example, in the Southern United States, an earlier planting date may allow flowering to complete before the worst of the typical late-summer heat and drought stress, but earlier planting may not be possible due to saturated spring soils. Earlier maturing hybrids may similarly escape late-season heat and drought but may sacrifice the yield potential of full-season hybrids. Other research has addressed the interaction between AF contamination and planting date (Damianidis et al. 2018) and the likelihood that climate change is shifting the suitable crop production windows, with corn production in the Southern United States to face substantial yield loss in coming years (Yang and Wang 2023). The late-season increase in airborne *A. flavus* inoculum observed in the present study is another factor for corn producers to consider in their management plans.

## Data Availability

No datasets were generated or analysed during the current study.

## Abbreviations

AF: Aflatoxin
mDRB: Modified Dichloran Rose-Bengal medium
PCoA: Principal coordinate analysis
CFU: Colony-forming units
